# Drug-induced headache reports: a comprehensive disproportionality and time-to-onset pharmacovigilance study using the FAERS database (2018–2024)

**DOI:** 10.3389/fpain.2025.1670648

**Published:** 2025-11-07

**Authors:** Abdulaziz Ibrahim Alzarea, Azfar Athar Ishaqui, Muhammad Bilal Maqsood, Abdullah Salah Alanazi, Aseel Awad Alsaidan, Tauqeer Hussain Mallhi, Narendar Kumar, Khalid M. Orayj, Sultan M. Alshahrani, Hassan H. Alhassan, Sami I. Alzarea, Omar Awad Alsaidan

**Affiliations:** 1 Department of Clinical Pharmacy, College of Pharmacy, Jouf University, Sakaka, Al-Jouf, Saudi Arabia; 2 Department of Clinical Pharmacy, College of Pharmacy, King Khalid University, Abha, Saudi Arabia; 3 Eastern Health Cluster, Ministry of Health, Dammam, Saudi Arabia; 4 Department of Family and Community Medicine, College of Medicine, Jouf University, Sakaka, Al-Jouf, Saudi Arabia; 5 Medicines R Us Chemist, Gregory Hills, NSW, Australia; 6 School of Pharmacy, Faculty of Health and Medical Sciences, Taylors University, Selangor, Malaysia; 7 Department of Pharmacy Practice, Faculty of Pharmacy, Sindh University, Jamshoro, Pakistan; 8 Department of Clinical Laboratory Sciences, College of Applied Medical Sciences, Jouf University, Sakaka, Al-Jouf, Saudi Arabia; 9 Department of Pharmacology, College of Pharmacy, Jouf University, Sakaka, Al-Jouf, Saudi Arabia; 10 Department of Pharmaceutics, College of Pharmacy, Jouf University, Sakaka, Al-Jouf, Saudi Arabia

**Keywords:** headache, drug safety, adverse drug reactions, disproportionality analysis, pharmacovigilance

## Abstract

**Background:**

Headache is a common adverse drug reaction (ADR) across diverse therapeutic classes, yet systematic evaluations of drug-associated headaches in real-world settings are limited. This study aimed to explore the association between various medications and the reporting of headache as an ADR using the FDA-Adverse Event Reporting System (FAERS).

**Methods:**

We conducted a retrospective disproportionality analysis using FAERS data from Q1-2018 to Q4-2024. Duplicate reports were removed per FDA guidelines. Reports with headache as an adverse event and drugs classified as Primary Suspect were included. Disproportionality metrics — Reporting Odds Ratio (ROR) and Proportional Reporting Ratio (PRR)—were calculated to identify signals. Drugs were classified according to the Anatomical Therapeutic Chemical(ATC) classification system, and time-to-onset analyses were performed.

**Results:**

A total of 313,166 headache-associated cases were identified. Females (66.66%) and patients aged 51–65 years (21.35%) were most commonly affected. The drugs with the highest headache risk based on ROR included glecaprevir/pibrentasvir (ROR = 10.445), sofosbuvir/velpatasvir (ROR = 9.729), and eptinezumab-jjmr (ROR = 6.775). Top frequently reported drugs were apremilast, treprostinil, and adalimumab. Calcium homeostasis agents (ROR = 6.268) and systemic antivirals (ROR = 4.259) emerged as the ATC classes with the highest headache signal strength. Early-onset headaches (≤7days) were particularly associated with ofatumumab and fingolimod. Late-onset headaches (>90days) were linked to treprostinil and infliximab-dyyb.

**Conclusion:**

This large-scale pharmacovigilance study identifies multiple drugs and therapeutic classes with significant associations to headache as an ADR. These findings highlight the need for proactive headache monitoring, particularly during early treatment phases, and warrant further prospective investigations to understand mechanisms and preventive strategies.

## 1 Introduction

Headache is a temporary or permanent functional disorder of the central nervous system, affecting a substantial proportion of the global population (1). It is estimated that nearly 50% of adults experience at least one headache per year (2), with a significant number progressing to chronic or disabling forms, such as migraine or tension-type headache (3). Beyond primary headache disorders, many cases are drug-induced, either as a direct adverse effect or due to medication overuse (4, 5). Medications targeting the central nervous system or cardiovascular system are commonly associated with the onset or aggravation of headaches (6–8).

The mechanisms behind drug-associated headaches (DAH) vary and often drug-specific. Some agents, such as nitrates, phosphodiesterase inhibitors, and calcium channel blockers, induce headaches through vasodilation and increased cerebral blood flow (9, 10). Others, including selective serotonin reuptake inhibitors (SSRIs) (11, 12) and hormonal therapies (13, 14), may alter neurotransmitter balance, leading to headache development. Additionally, analgesic overuse is a well-documented cause of medication-overuse headache (MOH), creating a vicious cycle of pain and dependency (15). Although evidence supports drug-induced headache as a recognized phenomenon, its detection in clinical practice remains inconsistent. Diagnostic ambiguity arises from overlapping symptom profiles and the inherent difficulties in isolating causative agents in patients with complex medication histories.

Current evidence on DAH primarily derives from case reports, observational studies, and clinical trials focused on specific drug classes, with limited systematic evaluation across broader therapeutic categories. We hypothesize that certain medications will show statistically significant disproportionality signals for headache compared to other drugs in the FDA Adverse Event Reporting System (FAERS) database. Therefore, current study aimed to address that gap by utilizing data from the FAERS and applying disproportionality analysis to explore the association between drug use and the reporting of headache as an adverse drug event (ADE). The study aims to identify drugs most frequently linked to headache reports, assess the strength of these as-sociations using statistical signal detection methods, and classify headache-related signals across various therapeutic classes based on the Anatomical Therapeutic Chemical (ATC) classification system. The current study's approach builds upon prior pharmacovigilance studies using FAERS data, such as Musialowicz et al. (16), which analyzed headache associations from 2018 to 2020 using reporting odds ratio (ROR) (16). By extending the timeframe to 2024, incorporating proportional reporting ratio (PRR) and ATC classification analyses, and including data on newly approved agents like CGRP monoclonal antibodies (e.g., galcanezumab and eptinezumab), this study provides an updated and expanded evaluation.

## 2 Material and methods

### 2.1 Data source

The FAERS served as the primary data source, providing access to patient demographics, drug exposure information, and spontaneously reported AEs. Our study period ranged from Quarter 1-2018 to Quarter 4-2024, identifying drugs and therapeutic classes most strongly associated with headache. The database was accessed via FDA's FAERS Public Dashboard (https://fis.fda.gov/extensions/FPD-QDE-FAERS/FPD-QDE-FAERS.html).

### 2.2 Data cleaning

**Figure 1:**
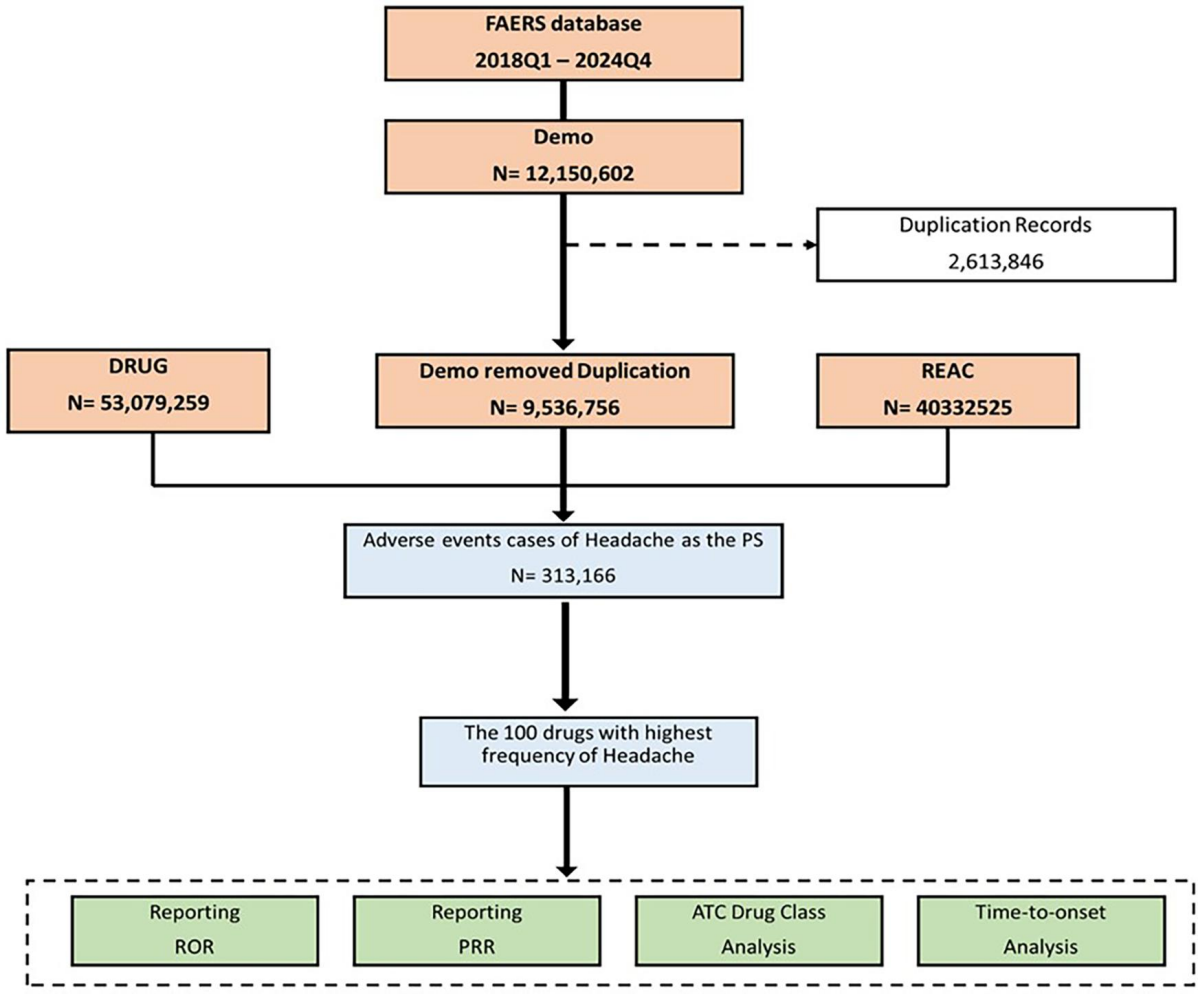
Flowchart of FAERS data selection and analysis for headache-related adverse events.

A total of 28 quarters of data, spanning from Q1 2018 to Q4 2024, was downloaded and stored in XML format. Each quarterly dataset included patient demographics (DEMO), indication (INDI), drug use records (DRUG), therapy duration (THER), adverse event records (REAC), and patient outcomes (OUTC). To process the downloaded data in R (version 4.3.3), the dataset was initially cleaned in accordance with FDA recommendations for eliminating duplicate reports. For reports sharing the same CASEID, the one with the latest FDA_DT value was preserved. In instances where both CASEID and FDA_DT were identical, the report with the highest PRIMARYID was retained. Subsequently, the dataset was further refined to include only reports where the target drug was designated as the Primary Suspect (PS), enhancing the reliability of the study. To reduce potential confounding, secondary suspect and interacting drugs were excluded from the background counts for ROR and PRR calculations (17). All drugs reported in association with headache were extracted. Both brand and generic names were standardized for consistency. The term “headache” was identified using the MedDRA preferred term. No subtypes (e.g., migraine, tension-type) were specifically analyzed in this study to maintain uniformity and minimize diagnostic bias in spontaneous reports. Further details of this process are illustrated in Figure 1.

### 2.3 Statistical analysis

The descriptive analyses in our study included frequencies and proportions of reported headaches stratified by drug class, and demographic variables. Statistical analysis was conducted using disproportionality methods, specifically the ROR and PRR, to evaluate associations between drug exposures and reported headache events within the FAERS database. For each drug and ATC class, ROR and PRR were calculated along with corresponding 95% confidence intervals (CIs), allowing for the identification of signals suggestive of a potential link to drug-associated headaches. The formula is used for computing ROR is as follows (18):

$$\mathrm{ROR}=\frac{a/b}{c/d}=\frac{a\cdot d}{b\cdot c}$$

The 95% CI for ROR is computed using the logarithmic transformation:

$$\mathrm{SE}(\ln\;(\mathrm{ROR}))=\sqrt{\frac{1}{a}+\frac{1}{b}+\frac{1}{c}+\frac{1}{d}}$$

95%CI=exp(ln(ROR)±1.96⋅SE(ln(ROR)))

The PRR measures the proportion of headache reports for the drug of interest relative to all drugs. The formula used for computing PRR is as follows (19):

$$\mathrm{PRR}=\frac{a/(a+b)}{c/(c+d)}$$

The 95% CI for PRR is similarly calculated using the logarithmic transformation:

$$\mathrm{SE}(\;\ln\;(\mathrm{PRR}))=\sqrt{\frac{1}{a}-\frac{1}{a+b}+\frac{1}{c}-\frac{1}{c+d}}$$

95%CI=exp(ln(PRR)±1.96⋅SE(ln(PRR)))

Where:

- a: Number of reports with the drug of interest and headache.
- b: Number of reports with the drug of interest and other adverse events.
- c: Number of reports with other drugs and headache.
- d: Number of reports with other drugs and other adverse events.

A drug was considered to have a positive signal if both ROR and PRR exceeded the threshold of 1.0 with the lower bound of the 95% CI also above 1 (20). These disproportionality measures provided a robust and standardized approach to detect elevated reporting frequencies of headache relative to other adverse events, supporting the identification of drugs and therapeutic categories with significant risk profiles. Onset time was calculated by subtracting the drug start date (start_dt) from the adverse event date (event_dt) in the FAERS database. Only reports with complete and valid date pairs were included.

## 3 Results

The analysis utilized data from the FAERS database spanning from the first quarter of 2018 to the fourth quarter of 2024. Initially, the dataset comprised 12,150,602 adverse event reports. After data cleansing, which included the removal of duplicate records (2,613,846 cases: approximately 21.5% of the initial dataset), the refined demographic dataset contained 9,536,756 unique entries. From these, a total of 313,166 cases were identified where headache was reported as the PS adverse event. The 100 drugs most frequently associated with headache were selected for further analysis, which included disproportionality assessments using the ROR and Proportional Reporting Ratio PRR, as well as an ATC drug class analysis and time-to-onset evaluation. Figure 1 summarizes the FAERS data extraction and analysis steps, from initial reports to the final selection of headache-associated drugs.

The data regarding demographic and reporting characteristics of headache-related adverse events is presented in Table 1. The data was comprised individuals across various age groups, with the majority aged 51–65 years (21.35%), followed by 31–50 years (18.14%) and >65 years (15.44%), while 36.57% had missing age data. Females constituted the majority (66.66%), with males accounting for 23.18%, and 10.05% had missing sex data. Regarding weight, most records were missing (73.32%), while 21.22% weighed >50–≤100 kg, and 3.37% weighed >100 kg. The consumers were the most frequent reporters (54.72%), followed by health professionals (15.93%) and medical doctors (14.90%). Geographically, the United States contributed the majority of reports (65.87%), followed by Canada (12.06%), the United Kingdom (4.12%), and Denmark (2.36%), with 12.95% originating from other countries.

**Table 1:** Demographic and reporting characteristics of headache-related adverse events.

Characteristics	Categorization	Frequency (*N*)	Percentage (%)
Age	<18 years	9,184	2.93
	18–30 years	17,472	5.58
	31–50 years	56,794	18.14
	51–65 years	66,862	21.35
	>65 years	48,343	15.44
	Missing	114,511	36.57
Sex	Female	208,746	66.66
	Male	72,601	23.18
	Missing	31,474	10.05
	Unknown	345	0.11
Weight	≤50 kg	6,533	2.09
	>50–≤100 kg	66,462	21.22
	>100 kg	10,548	3.37
	Missing	229,623	73.32
Occupation Reporter	Consumer	171,354	54.72
	Health Professional	49,874	15.93
	Medical Doctor	46,659	14.90
	Pharmacist	16,296	5.20
	Other Work	1,601	0.51
	Others	20,337	6.49
	Not Available	7,045	2.25
Reporter Country	United States	206,270	65.87
	Canada	37,782	12.06
	United Kingdom	12,904	4.12
	Denmark	7,381	2.36
	Country Not Specified	8,271	2.64
	Others	40,558	12.95

Table 2 presents Frequency, RORs and PRR of top 100 drugs Associated with headache. Among the drugs analyzed for headache as an adverse drug reaction, glecaprevir/pibrentasvir exhibited the highest ROR at 10.445 (95% CI: 10.001–10.908), indicating a strong association with the reported outcome. This was closely followed by sofosbuvir/velpatasvir with an ROR of 9.729 (95% CI: 9.338–10.137), and eptinezumab-jjmr with an ROR of 6.775 (95% CI: 6.302–7.283). Other drugs with notably high RORs included abaloparatide (6.268), ledipasvir/sofosbuvir (5.937), and galcanezumab-gnlm (4.251), suggesting a relatively higher likelihood of reporting associated adverse events. On the opposite end of the spectrum, acetaminophen showed one of the lowest RORs at 0.05, followed closely by cetirizine hydrochloride (0.062), leflunomide (0.076), and metformin (0.078), reflecting a comparatively lower signal for adverse event reporting.

**Table 2:** Frequency, reporting odds ratios (RORs) and proportional reporting ratios of top 100 drugs associated with headache.

Drug	Frequency	ROR (95% CI)^1^	PRR (95% CI)^1^	ADR status mentioned in drug profile	ADR frequency IN drug profile
APREMILAST	8,694	2.021 (1.978–2.065)	2.006 (1.963–2.049)	YES	>10%
TREPROSTINIL	7,014	2.387 (2.331–2.445)	2.362 (2.307–2.419)	YES	>10%
ADALIMUMAB	6,981	0.349 (0.34–0.357)	0.35 (0.342–0.359)	YES	>10%
TOFACITINIB CITRATE	6,594	0.536 (0.523–0.549)	0.538 (0.525–0.551)	YES	1% to 10%
DUPILUMAB	5,799	0.786 (0.766–0.807)	0.788 (0.767–0.808)	YES	1% to 10%
HUMAN IMMUNOGLOBULIN G	5,725	1.345 (1.31–1.381)	1.341 (1.306–1.377)	YES	>10%
OFATUMUMAB	4,314	3.077 (2.985–3.172)	3.029 (2.938–3.122)	YES	>10%
FINGOLIMOD HYDROCHLORIDE	4,085	2.132 (2.067–2.2)	2.114 (2.049–2.181)	YES	>10%
SECUKINUMAB	3,463	0.38 (0.367–0.393)	0.382 (0.369–0.395)	YES	1% to 10%
METHOTREXATE	3,188	0.147 (0.142–0.152)	1.066 (1.029–1.104)	YES	1% to 10%
MACITENTAN	3,145	1.067 (1.03–1.105)	6.024 (5.808–6.247)	YES	>10%
ABALOPARATIDE	3,072	6.268 (6.044–6.5)	0.243 (0.235–0.253)	YES	1% to 10%
RITUXIMAB	3,064	0.212 (0.205–0.22)	1.019 (0.983–1.057)	YES	1% to 10%
INFLIXIMAB	3,062	0.242 (0.233–0.251)	1.914 (1.843–1.987)	YES	>10%
OCRELIZUMAB	2,999	1.02 (0.983–1.057)	1.393 (1.342–1.987)	YES	>10%
SELEXIPAG	2,818	1.927 (1.857–2.001)	0.213 (0.206–0.222)	YES	>10%
NIRAPARIB	2,795	1.397 (1.346–1.45)	0.574 (0.553–0.597)	YES	>10%
OMALIZUMAB	2,611	0.572 (0.551–0.595)	0.489 (0.47–0.509)	YES	>10%
LENALIDOMIDE	2,543	0.487 (0.469–0.507)	0.236 (0.227–0.246)	YES	>10%
LEVOTHYROXINE SODIUM	2,476	0.235 (0.226–0.244)	9.116 (8.749–9.498)	YES	FREQUENCY NOT DEFINED
SOFOSBUVIR\VELPATASVIR	2,472	9.729 (9.338–10.137)	0.561 (0.539–0.584)	YES	>10%
VEDOLIZUMAB	2,421	0.559 (0.537–0.582)	0.216 (0.208–0.225)	YES	>10%
ABATACEPT	2,399	0.215 (0.206–0.224)	0.474 (0.455–0.493)	YES	>10%
INFLIXIMAB-DYYB	2,382	0.472 (0.453–0.491)	9.322 (9.322–10.167)	YES	>10%
GLECAPREVIR\PIBRENTASVIR	2,219	10.445 (10.001–10.908)	0.143 (0.143–0.153)	YES	>10%
EVOLOCUMAB	2,138	0.773 (0.741–0.807)	0.742 (0.742–0.808)	YES	>10%
PALBOCICLIB	2,116	0.649 (0.621–0.677)	0.623 (0.623–0.679)	NO	-
GALCANEZUMAB-GNLM	2,094	4.251 (4.07–4.441)	3.97 (3.97–4.332)	NO	-
ETANERCEPT	2,071	0.155 (0.149–0.162)	0.15 (0.15–0.163)	POST MARKETING	-
TOCILIZUMAB	2,048	0.167 (0.16–0.175)	0.161 (0.161–0.176)	YES	1% to 10%
NIRMATRELVIR\RITONAVIR	2,012	1.71 (1.637–1.788)	1.628 (1.628–1.778)	POST MARKETING	-
LEVONORGESTREL	1,982	1.424 (1.362–1.489)	1.358 (1.358–1.483)	YES	>10%
ERENUMAB-AOOE	1,980	1.19 (1.139–1.244)	1.137 (1.137–1.242)	NO	-
CERTOLIZUMAB PEGOL	1,956	0.228 (0.218–0.238)	0.219 (0.219–0.239)	YES	1% to 10%
TERIFLUNOMIDE	1,780	1.573 (1.501–1.649)	1.495 (1.495–1.641)	YES	>10%
VOXELOTOR	1,770	3.215 (3.067–3.371)	3.015 (3.015–3.314)	YES	>10%
DIMETHYL FUMARATE	1,769	1.049 (1.001–1.099)	1 (1–1.099)	NO	-
PREGABALIN	1,756	0.178 (0.17–0.187)	0.171 (0.171–0.188)	YES	>10%
RUXOLITINIB	1,658	0.722 (0.688–0.758)	0.69 (0.69–0.76)	YES	>10%
SEMAGLUTIDE	1,657	0.915 (0.872–0.961)	0.873 (0.873–0.961)	YES	>10%
AMBRISENTAN	1,570	0.883 (0.841–0.928)	0.841 (0.841–0.929)	YES	>10%
SOMATROPIN	1,531	0.981 (0.933–1.032)	0.933 (0.933–1.032)	YES	>10%
SODIUM OXYBATE	1,522	1.043 (0.992–1.097)	0.991 (0.991–1.097)	YES	>10%
ONABOTULINUMTOXINA	1,477	1.292 (1.227–1.36)	1.224 (1.224–1.357)	YES	>10%
NATALIZUMAB	1,455	1.166 (1.107–1.228)	1.106 (1.106–1.226)	YES	>10%
APIXABAN	1,397	0.208 (0.197–0.219)	0.199 (0.199–0.221)	NO	-
AMLODIPINE BESYLATE	1,380	0.109 (0.104–0.115)	0.104 (0.104–0.116)	NO	-
ACETAMINOPHEN	1,377	0.05 (0.048–0.053)	0.048 (0.048–0.053)	YES	1% to 10%
OCTREOTIDE ACETATE	1,365	0.462 (0.438–0.487)	0.44 (0.44–0.489)	YES	>10%
AMPHETAMINE ASPARTATE\AMPHETAMINE SULFATE\DEXTROAMPHETAMINE SACCHARATE\DEXTROAMPHETAMINE SULFATE	1,361	0.972 (0.921–1.025)	0.922 (0.922–1.026)	YES	>10%
RISANKIZUMAB-RZAA	1,349	1.106 (1.048–1.166)	1.064 (1.064–1.183)	YES	1% to 10%
USTEKINUMAB	1,343	0.241 (0.229–0.255)	0.23 (0.23–0.256)	YES	1% to 10%
MEPOLIZUMAB	1,323	0.44 (0.416–0.464)	0.418 (0.418–0.466)	YES	>10%
SACUBITRIL\VALSARTAN	1,310	0.413 (0.391–0.436)	0.393 (0.393–0.438)	NO	-
ECULIZUMAB	1,289	1.745 (1.652–1.844)	1.642 (1.642–1.833)	YES	>10%
INTERFERON BETA-1A	1,215	1.03 (0.973–1.09)	0.973 (0.973–1.09)	YES	>10%
IBUPROFEN	1,213	0.117 (0.11–0.124)	0.111 (0.111–0.125)	YES	1% to 10%
UPADACITINIB	1,182	0.703 (0.664–0.744)	0.665 (0.665–0.746)	YES	1% to 10%
ALEMTUZUMAB	1,132	1.002 (0.945–1.063)	0.945 (0.945–1.062)	YES	>10%
EPOPROSTENOL	1,123	1.399 (1.319–1.484)	1.315 (1.315–1.48)	YES	>10%
ISOTRETINOIN	1,119	2.365 (2.229–2.509)	2.205 (2.205–2.483)	POST MARKETING	-
MINOXIDIL	1,108	1.559 (1.469–1.654)	1.463 (1.463–1.479)	YES	>10%
DALFAMPRIDINE	1,088	0.742 (0.699–0.787)	0.7 (0.7–0.789)	YES	1% to 10%
SERTRALINE	1,073	0.198 (0.187–0.211)	0.188 (0.188–0.225)	NO	-
TADALAFIL	1,060	0.422 (0.397–0.448)	0.399 (0.399–0.45)	YES	>10%
LEDIPASVIR\SOFOSBUVIR	1,014	5.937 (5.574–6.323)	5.369 (5.369–6.09)	YES	>10%
IBRUTINIB	1,008	0.522 (0.49–0.555)	0.492 (0.492–0.557)	YES	>10%
NINTEDANIB	986	0.773 (0.726–0.823)	0.728 (0.728–0.825)	YES	1% to 10%
GABAPENTIN	974	0.09 (0.085–0.096)	0.085 (0.085–0.097)	NO	-
TIRZEPATIDE	967	0.851 (0.799–0.907)	0.8 (0.8–0.908)	NO	-
ESTRADIOL	963	0.437 (0.41–0.466)	0.412 (0.412–0.467)	YES	>10%
VENLAFAXINE	953	0.23 (0.216–0.245)	0.217 (0.217–0.246)	YES	<1%
BELIMUMAB	943	1.126 (1.056–1.201)	1.055 (1.055–1.199)	NO	-
DULAGLUTIDE	943	0.61 (0.572–0.65)	0.574 (0.574–0.652)	NO	-
NALTREXONE	921	1.337 (1.253–1.427)	0.249 (0.249–0.254)	YES	>10%
METFORMIN	881	0.078 (0.073–0.083)	0.073 (0.073–0.107)	YES	1% to 10%
LEFLUNOMIDE	879	0.076 (0.071–0.082)	0.072 (0.072–0.082)	YES	>10%
SIPONIMOD	871	2.547 (2.382–2.724)	2.354 (2.354–2.692)	YES	>10%
CIPROFLOXACIN	865	0.299 (0.279–0.319)	0.281 (0.281–0.321)	YES	1% to 10%
DASATINIB	860	1.625 (1.519–1.738)	1.512 (1.512–1.73)	YES	>10%
ZOLEDRONIC ACID	860	0.404 (0.377–0.432)	0.379 (0.379–0.434)	YES	>10%
PIRFENIDONE	847	1.083 (1.012–1.159)	1.012 (1.012–1.089)	YES	>10%
OXYCODONE HYDROCHLORIDE	845	0.111 (0.104–0.119)	0.104 (0.104–0.119)	YES	>10%
ELEXACAFTOR\IVACAFTOR\TEZACAFTOR	823	2.203 (2.056–2.36)	2.037 (2.037–2.338)	YES	>10%
DENOSUMAB	786	0.237 (0.221–0.254)	0.222 (0.222–0.255)	YES	>10%
RIOCIGUAT	783	0.652 (0.607–0.699)	0.609 (0.609–0.701)	YES	>10%
NIVOLUMAB	781	0.301 (0.28–0.323)	0.218 (0.218–0.255)	YES	>10%
EPTINEZUMAB-JJMR	773	6.775 (6.302–7.283)	6.469 (6.469–7.443)	NO	-
LENVATINIB	766	0.909 (0.847–0.976)	0.847 (0.847–0.977)	YES	>10%
ETONOGESTREL	765	1.036 (0.965–1.113)	0.965 (0.965–1.113)	YES	>10%
RIVAROXABAN	764	0.191 (0.178–0.205)	0.179 (0.179–0.207)	NO	-
RIBOCICLIB	756	0.434 (0.404–0.466)	0.406 (0.406–0.468)	YES	>10%
MONTELUKAST SODIUM	736	0.1 (0.093–0.107)	0.093 (0.093–0.108)	YES	1% to 10%
ENZALUTAMIDE	734	0.737 (0.685–0.792)	0.687 (0.687–0.794)	YES	>10%
CABOZANTINIB S-MALATE	734	0.646 (0.601–0.694)	0.602 (0.602–0.794)	YES	>10%
CETIRIZINE HYDROCHLORIDE	679	0.062 (0.057–0.066)	0.058 (0.058–0.097)	YES	>10%
ARIPIPRAZOLE	660	0.228 (0.211–0.246)	0.213 (0.213–0.245)	YES	>10%
BENRALIZUMAB	655	1.543 (1.428–1.667)	1.422 (1.422–1.66)	YES	>10%
TOPIRAMATE	654	0.234 (0.217–0.253)	0.218 (0.218–0.255)	YES	FREQUENCY NOT DEFINED
POMALIDOMIDE	647	0.449 (0.416–0.485)	0.417 (0.417–0.487)	YES	>10%

Footnote 1: Signals defined as ROR/PRR >1 with lower 95% CI >1.

The top 10 most frequently reported drugs associated with headache, based on adverse event report frequency, were led by apremilast with 8,694 reports, making it the most commonly implicated agent. This was followed by treprostinil (7,014) and adalimumab (6,981). Tofacitinib citrate ranked fourth with 6,594 reports, closely followed by dupilumab (5,799) and human immunoglobulin G (5,725). The list also included ofatumumab (4,314), fingolimod hydrochloride (4,085), secukinumab (3,463), and methotrexate (3,188).

**Figure 2:**
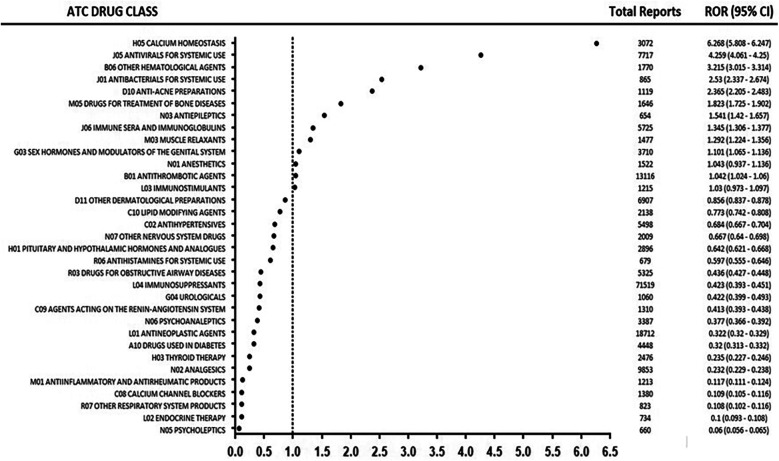
Signal strength (ROR) of the top drug classes associated with headache reports, stratified by ATC classification.

The pharmacovigilance analysis, stratified by ATC classification (Table 3), revealed significant variations in the association between drug classes and headache as an adverse drug reaction (ADR). Calcium homeostasis agents showed the strongest risk (ROR = 6.268), followed by systemic antivirals (ROR = 4.259), other hematological agents (ROR = 3.215), systemic antibacterials (ROR = 2.53), and anti-acne preparations (ROR = 2.365). Intermediate risks were observed for drugs treating bone diseases (ROR = 1.823), antiepileptics (ROR = 1.541), and sex hormones and modulators of the genital system (ROR = 1.101). The analysis revealed several noteworthy patterns regarding reporting frequency and risk magnitude. Immunosuppressants accounted for the highest number of reports (*n* = 28) yet demonstrated low risk (ROR = 0.423), while Antineoplastic agents, despite frequent reporting (*n* = 12), similarly showed weak association (ROR = 0.322). Cardiovascular agents including Calcium channel blockers and Antihypertensives displayed consistently low risk profiles. Figure 2 summarizes the ATC class signal values for the top drugs linked to headache reports.

**Table 3:** Association between drug classes (ATC classification) and headache as an adverse drug reaction (ADR), presented by reporting odds ratio (ROR) and frequency.

ATC	No. of drugs	ROR (95% CI)	PRR (95% CI)
A10 DRUGS USED IN DIABETES	4	0.32 (0.313–0.332)	0.322 (0.313–0.332)
B01 ANTITHROMBOTIC AGENTS	5	1.042 (1.024–1.06)	1.042 (1.024–1.06)
B06 OTHER HEMATOLOGICAL AGENTS	1	3.215 (3.015–3.314)	3.161 (3.015–3.314)
C02 ANTIHYPERTENSIVES	3	0.684 (0.667–0.704)	0.685 (0.667–0.704)
C08 CALCIUM CHANNEL BLOCKERS	1	0.109 (0.105–0.116)	0.11 (0.105–0.116)
C09 AGENTS ACTING ON THE RENIN-ANGIOTENSIN SYSTEM	1	0.413 (0.393–0.438)	0.415 (0.393–0.438)
C10 LIPID MODIFYING AGENTS	1	0.773 (0.742–0.808)	0.774 (0.742–0.808)
D10 ANTI-ACNE PREPARATIONS	1	2.365 (2.205–2.483)	2.34 (2.205–2.483)
D11 OTHER DERMATOLOGICAL PREPARATIONS	2	0.856 (0.837–0.878)	0.857 (0.837–0.877)
G03 SEX HORMONES AND MODULATORS OF THE GENITAL SYSTEM	3	1.101 (1.065–1.136)	1.1 (1.065–1.137)
G04 UROLOGICALS	1	0.422 (0.399–0.493)	0.424 (0.399–0.493)
H01 PITUITARY AND HYPOTHALAMIC HORMONES AND ANALOGUES	2	0.642 (0.621–0.668)	0.644 (0.621–0.668)
H03 THYROID THERAPY	1	0.235 (0.227–0.246)	0.236 (0.227–0.246)
H05 CALCIUM HOMEOSTASIS	1	6.268 (5.808–6.247)	6.024 (5.808–6.247)
J01 ANTIBACTERIALS FOR SYSTEMIC USE	1	2.53 (2.337–2.674)	2.5 (2.337–2.674)
J05 ANTIVIRALS FOR SYSTEMIC USE	4	4.259 (4.061–4.25)	4.155 (4.061–4.25)
J06 IMMUNE SERA AND IMMUNOGLOBULINS	1	1.345 (1.306–1.377)	1.341 (1.306–1.377)
L01 ANTINEOPLASTIC AGENTS	12	0.322 (0.32–0.329)	0.324 (0.32–0.329)
L02 ENDOCRINE THERAPY	1	0.1 (0.093–0.108)	0.093 (0.093–0.108)
L03 IMMUNOSTIMULANTS	1	1.03 (0.973–1.097)	1.03 (0.973–1.097)
L04 IMMUNOSUPPRESSANTS	28	0.423 (0.393–0.451)	0.425 (0.422–0.451)
M01 ANTIINFLAMMATORY AND ANTIRHEUMATIC PRODUCTS	1	0.117 (0.111–0.124)	0.111 (0.111–0.124)
M03 MUSCLE RELAXANTS	1	1.292 (1.224–1.356)	1.289 (1.224–1.356)
M05 DRUGS FOR TREATMENT OF BONE DISEASES	2	1.823 (1.725–1.902)	1.812 (1.725–1.902)
N01 ANESTHETICS	1	1.043 (0.937–1.136)	1.043 (0.991–1.097)
N02 ANALGESICS	7	0.232 (0.229–0.238)	0.234 (0.229–0.238)
N03 ANTIEPILEPTICS	1	1.541 (1.42–1.657)	1.534 (1.42–1.657)
N05 PSYCHOLEPTICS	1	0.06 (0.056–0.065)	0.06 (0.056–0.065)
N06 PSYCHOANALEPTICS	3	0.377 (0.366–0.392)	0.379 (0.366–0.392)
N07 OTHER NERVOUS SYSTEM DRUGS	2	0.667 (0.64–0.698)	0.668 (0.64–0.698)
R03 DRUGS FOR OBSTRUCTIVE AIRWAY DISEASES	4	0.436 (0.427–0.448)	0.438 (0.427–0.448)
R06 ANTIHISTAMINES FOR SYSTEMIC USE	1	0.597 (0.555–0.646)	0.556 (0.556–0.646)
R07 OTHER RESPIRATORY SYSTEM PRODUCTS	1	0.108 (0.102–0.116)	0.102 (0.102–0.116)

Table 4 presents headache onset timing across different drug classes. Ofatumumab. abaloparatide and fingolimod hydrochloride exhibited the highest proportion of early-onset headaches (≤7 days), reported in 78.6%, 69.5% and 58.5% of cases, respectively. In contrast, treprostinil and infliximab-dyyb showed delayed onset patterns, with 59% of headaches occurring after 90 days. Moreover, nirmatrelvir/ritonavir demonstrated a sharp decline in headache reports beyond the first week, with only 5.4% occurring after 90 days. Among immunosuppressants, vedolizumab and adalimumab had substantial late-onset cases (47% and 53.2%, respectively. Dupilumab and macitentan displayed intermediate distributions, with 37.9% and 51.3% of headaches emerging after 90 days.

**Table 4:** Time-to-onset distribution of the Top 50 drugs associated with drug-induced headache based on total complete cases.

Drug name	ATC	Total complete cases	≤ 7 days	8-28 days	29-60 days	61-90 days	>90 days
APREMILAST	L04 IMMUNOSUPPRESSANTS	2,552	1,171 (45.90%)	342 (13.5%)	201 (7.90%)	90 (3.50%)	748 (29.30%)
TREPROSTINIL	B01 ANTITHROMBOTIC AGENTS	2,352	375 (15.90%)	326 (13.90%)	153 (6.50%)	110 (4.70%)	1,388 (59.00%)
FINGOLIMOD HYDROCHLORIDE	L04 IMMUNOSUPPRESSANTS	2,261	1,323 (58.50%)	167 (7.40%)	110 (4.90%)	64 (2.80%)	597 (26.40%)
HUMAN IMMUNOGLOBULIN G	J06 IMMUNE SERA AND IMMUNOGLOBULINS	2,104	1,030 (49.00%)	166 (7.90%)	103 (4.90%)	72 (3.40%)	733 (34.80%)
OFATUMUMAB	L04 IMMUNOSUPPRESSANTS	2,014	1,582 (78.60%)	183 (9.00%)	75 (3.70%)	21 (1.00%)	153 (7.60%)
INFLIXIMAB-DYYB	L04 IMMUNOSUPPRESSANTS	1,912	292 (15.30%)	219 (11.50%)	168 (8.80%)	104 (5.40%)	1,129 (59.00%)
OCRELIZUMAB	L04 IMMUNOSUPPRESSANTS	1,729	947 (54.80%)	156 (9.00%)	46 (2.70%)	20 (1.20%)	560 (32.40%)
DUPILUMAB	D11 OTHER DERMATOLOGICAL PREPARATIONS	1,678	574 (34.20%)	238 (14.10%)	137 (8.20%)	93 (5.50%)	636 (37.90%)
ADALIMUMAB	L04 IMMUNOSUPPRESSANTS	1,627	410 (25.20%)	178 (10.90%)	106 (6.50%)	68 (4.20%)	865 (53.20%)
NIRMATRELVIR\RITONAVIR	J05 ANTIVIRALS FOR SYSTEMIC USE	1,510	796 (52.70%)	617 (40.80%)	13 (0.90%)	2 (0.10%)	82 (5.40%)
VEDOLIZUMAB	L04 IMMUNOSUPPRESSANTS	1,395	418 (30.00%)	141 (10.00%)	104 (7.50%)	77 (5.50%)	655 (47.00%)
TOFACITINIB CITRATE	L04 IMMUNOSUPPRESSANTS	1,345	422 (31.40%)	175 (13.00%)	93 (6.90%)	70 (5.20%)	585 (43.50%)
OMALIZUMAB	R03 DRUGS FOR OBSTRUCTIVE AIRWAY DISEASES	1,312	410 (31.20%)	106 (8.10%)	94 (7.20%)	54 (4.10%)	648 (49.40%)
ABALOPARATIDE	H05 CALCIUM HOMEOSTASIS	1,284	893 (69.50%)	182 (14.20%)	62 (4.80%)	32 (2.50%)	115 (9.00%)
MACITENTAN	C02 ANTIHYPERTENSIVES	1,224	261 (21.30%)	182 (14.90%)	108 (8.80%)	45 (3.70%)	628 (51.30%)
SELEXIPAG	B01 ANTITHROMBOTIC AGENTS	1,166	415 (35.60%)	235 (20.10%)	119 (10.20%)	60 (5.10%)	337 (28.90%)
SECUKINUMAB	L04 IMMUNOSUPPRESSANTS	1,137	380 (33.40%)	167 (14.70%)	103 (9.10%)	53 (4.70%)	434 (38.20%)
NIRAPARIB	L01 ANTINEOPLASTIC AGENTS	1,036	384 (37.10%)	313 (30.20%)	89 (8.60%)	50 (4.80%)	200 (19.30%)
LEVONORGESTREL	G03 SEX HORMONES AND MODULATORS OF THE GENITAL SYSTEM	980	642 (65.50%)	91 (9.30%)	34 (3.50%)	10 (1.00%)	203 (20.70%)
GLECAPREVIR\PIBRENTASVIR	J05 ANTIVIRALS FOR SYSTEMIC USE	890	300 (33.70%)	274 (30.80%)	127 (14.30%)	22 (2.50%)	167 (18.80%)
CERTOLIZUMAB PEGOL	L04 IMMUNOSUPPRESSANTS	762	284 (37.30%)	77 (10.10%)	73 (9.60%)	37 (4.90%)	291 (38.20%)
SOFOSBUVIR\VELPATASVIR	J05 ANTIVIRALS FOR SYSTEMIC USE	744	264 (35.50%)	192 (25.90%)	115 (15.50%)	18 (2.40%)	155 (20.80%)
NATALIZUMAB	L04 IMMUNOSUPPRESSANTS	732	293 (40.00%)	68 (9.30%)	26 (3.60%)	12 (1.60%)	333 (45.50%)
OCTREOTIDE ACETATE	H01 PITUITARY AND HYPOTHALAMIC HORMONES AND ANALOGUES	679	146 (21.50%)	47 (7.00%)	32 (4.70%)	35 (5.20%)	419 (61.70%)
SEMAGLUTIDE	A10 DRUGS USED IN DIABETES	666	404 (60.70%)	95 (14.40%)	50 (7.50%)	17 (2.60%)	100 (15.00%)
VOXELOTOR	B06 OTHER HEMATOLOGICAL AGENTS	661	218 (33.00%)	125 (18.90%)	43 (6.50%)	44 (6.70%)	231 (34.90%)
ONABOTULINUMTOXINA	M03 MUSCLE RELAXANTS	622	416 (66.90%)	62 (9.90%)	16 (2.60%)	8 (1.30%)	120 (19.30%)
RUXOLITINIB	L01 ANTINEOPLASTIC AGENTS	616	215 (34.90%)	106 (17.20%)	69 (11.20%)	18 (2.90%)	208 (33.80%)
PALBOCICLIB	L01 ANTINEOPLASTIC AGENTS	595	114 (19.20%)	109 (18.30%)	64 (10.80%)	35 (5.90%)	273 (45.90%)
LENALIDOMIDE	L04 IMMUNOSUPPRESSANTS	576	142 (24.70%)	82 (14.20%)	46 (8.00%)	31 (5.40%)	275 (47.70%)
RISANKIZUMAB-RZAA	L04 IMMUNOSUPPRESSANTS	571	125 (21.90%)	86 (15.10%)	34 (6.00%)	29 (5.10%)	297 (52.00%)
MEPOLIZUMAB	R03 DRUGS FOR OBSTRUCTIVE AIRWAY DISEASES	557	192 (34.50%)	41 (7.40%)	49 (8.80%)	13 (2.30%)	262 (47.00%)
NINTEDANIB	L01 ANTINEOPLASTIC AGENTS	552	197 (35.70%)	123 (22.30%)	55 (10.00%)	26 (4.70%)	151 (27.40%)
ABATACEPT	L04 IMMUNOSUPPRESSANTS	551	155 (28.10%)	64 (11.60%)	35 (6.40%)	23 (4.20%)	274 (49.70%)
ALEMTUZUMAB	L04 IMMUNOSUPPRESSANTS	551	388 (70.40%)	22 (4.00%)	8 (1.50%)	4 (0.70%)	129 (23.40%)
ACETAMINOPHEN	N02 ANALGESICS	550	477 (86.70%)	19 (3.50%)	8 (1.50%)	6 (1.10%)	40 (7.30%)
DIMETHYL FUMARATE	L04 IMMUNOSUPPRESSANTS	526	226 (43.00%)	79 (15.00%)	29 (5.50%)	5 (1.00%)	187 (35.60%)
AMPHETAMINE \DEXTROAMPHETAMINE	N06 PSYCHOANALEPTICS	522	414 (79.30%)	50 (9.50%)	24 (4.60%)	6 (1.10%)	28 (5.40%)
AMBRISENTAN	C02 ANTIHYPERTENSIVES	503	82 (16.30%)	41 (8.20%)	45 (8.90%)	21 (4.20%)	314 (62.40%)
NIVOLUMAB	L01 ANTINEOPLASTIC AGENTS	485	154 (31.80%)	123 (25.40%)	71 (14.60%)	42 (8.70%)	95 (19.60%)
UPADACITINIB	L04 IMMUNOSUPPRESSANTS	477	108 (22.60%)	58 (12.20%)	43 (9.00%)	32 (6.70%)	236 (49.50%)
TERIFLUNOMIDE	L04 IMMUNOSUPPRESSANTS	464	101 (21.80%)	63 (13.50%)	43 (9.30%)	24 (5.20%)	233 (50.20%)
LEVOTHYROXINE SODIUM	H03 THYROID THERAPY	445	196 (44.00%)	85 (19.10%)	64 (14.40%)	20 (4.50%)	80 (18.00%)
AMLODIPINE BESYLATE	C08 CALCIUM CHANNEL BLOCKERS	436	255 (58.50%)	59 (13.60%)	25 (5.70%)	9 (2.10%)	88 (20.20%)
EVOLOCUMAB	C10 LIPID MODIFYING AGENTS	420	219 (52.10%)	67 (16.00%)	22 (5.20%)	24 (5.70%)	88 (21.00%)
IBUPROFEN	M01 ANTIINFLAMMATORY AND ANTIRHEUMATIC PRODUCTS	411	296 (72.00%)	22 (5.30%)	23 (5.60%)	14 (3.40%)	56 (13.60%)
ZOLEDRONIC ACID	M05 DRUGS FOR TREATMENT OF BONE DISEASES	410	344 (83.90%)	13 (3.20%)	10 (2.40%)	7 (1.70%)	36 (8.80%)
CIPROFLOXACIN	J01 ANTIBACTERIALS FOR SYSTEMIC USE	407	283 (69.50%)	60 (14.80%)	17 (4.20%)	10 (2.50%)	37 (9.10%)
SIPONIMOD	L04 IMMUNOSUPPRESSANTS	404	287 (71.00%)	36 (8.90%)	23 (5.70%)	9 (2.20%)	49 (12.10%)
ERENUMAB-AOOE	N02 ANALGESICS	400	215 (53.80%)	40 (9.90%)	34 (8.50%)	25 (6.20%)	86 (21.50%)

The analysis of headache onset timelines across different ATC drug categories is presented in Table 5. It was revealed that the drugs used in diabetes (A10) and anti-inflammatory/antirheumatic agents (M01) had the highest proportion of early-onset headaches (≤7 days), occurring in 64.5% and 72% of cases, respectively. Similarly, calcium homeostasis agents (H05) and antibacterials (J01) showed rapid onset, with 69.5% of headaches reported within the first week. In contrast, antithrombotic agents (B01) and pituitary/hypothalamic hormones (H01) exhibited delayed reactions, with 47% and 52.1% of headaches occurring after 90 days. Immunosuppressants (L04), despite being the most frequently reported drug class (*n* = 24,017), demonstrated a balanced distribution, with 41% early-onset and 37.8% delayed-onset headaches. Antivirals (J05) displayed a unique bimodal pattern, with 42.6% early-onset and a sharp decline after the first month, while immunostimulants (L03) had the highest delayed-onset rate (62.5% after 90 days).

**Table 5:** Time-to-onset distribution of the Top 100 drugs associated with drug-induced headache by ATC category in total complete cases.

ATC Category	No. of drugs	Total complete caseS	≤ 7 days	8-28 days	29-60 days	61–90 days	>90 days
A10 DRUGS USED IN DIABETES	3	1,077	695 (64.5%)	132 (12.3%)	65 (6%)	25 (2.3%)	160 (14.9%)
B01 ANTITHROMBOTIC AGENTS	5	4,396	1,125 (25.6%)	670 (15.2%)	337 (7.7%)	200 (4.5%)	2,064 (47%)
B06 OTHER HEMATOLOGICAL AGENTS	3	661	218 (33%)	125 (18.9%)	43 (6.5%)	44 (6.7%)	231 (34.9%)
C02 ANTIHYPERTENSIVES	3	2,002	398 (19.9%)	264 (13.2%)	179 (8.9%)	91 (4.5%)	1,070 (53.4%)
C08 CALCIUM CHANNEL BLOCKERS	1	436	255 (58.5%)	59 (13.5%)	25 (5.7%)	9 (2.1%)	88 (20.2%)
C09 AGENTS ACTING ON THE RENIN-ANGIOTENSIN SYSTEM	1	272	115 (42.3%)	50 (18.4%)	20 (7.4%)	12 (4.4%)	75 (27.6%)
C10 LIPID MODIFYING AGENTS	1	420	219 (52.1%)	67 (16%)	22 (5.2%)	24 (5.7%)	88 (21%)
D10 ANTI-ACNE PREPARATIONS	1	206	67 (32.5%)	35 (17%)	25 (12.1%)	19 (9.2%)	60 (29.1%)
D11 OTHER DERMATOLOGICAL PREPARATIONS	2	1,944	756 (38.9%)	251 (12.9%)	141 (7.3%)	94 (4.8%)	702 (36.1%)
G03 SEX HORMONES AND MODULATORS OF THE GENITAL SYSTEM	3	1,493	910 (61%)	131 (8.8%)	66 (4.4%)	25 (1.7%)	361 (24.2%)
G04 UROLOGICALS	1	381	131 (34.4%)	48 (12.6%)	37 (9.7%)	20 (5.2%)	145 (38.1%)
H01 PITUITARY AND HYPOTHALAMIC HORMONES AND ANALOGUES	2	1,043	300 (28.8%)	96 (9.2%)	53 (5.1%)	51 (4.9%)	543 (52.1%)
H03 THYROID THERAPY	1	445	196 (44%)	85 (19.1%)	64 (14.4%)	20 (4.5%)	80 (18%)
H05 CALCIUM HOMEOSTASIS	1	1,284	893 (69.5%)	182 (14.2%)	62 (4.8%)	32 (2.5%)	115 (9%)
J01 ANTIBACTERIALS FOR SYSTEMIC USE	1	407	283 (69.5%)	60 (14.7%)	17 (4.2%)	10 (2.5%)	37 (9.1%)
J05 ANTIVIRALS FOR SYSTEMIC USE	4	3,498	1,490 (42.6%)	1,187 (33.9%)	307 (8.8%)	54 (1.5%)	460 (13.2%)
J06 IMMUNE SERA AND IMMUNOGLOBULINS	1	2,104	1,030 (49%)	166 (7.9%)	103 (4.9%)	72 (3.4%)	733 (34.8%)
L01 ANTINEOPLASTIC AGENTS	12	5,256	1,671 (31.8%)	1,139 (21.7%)	526 (10%)	269 (5.1%)	1,651 (31.4%)
L02 ENDOCRINE THERAPY	1	241	75 (31.1%)	43 (17.8%)	21 (8.7%)	13 (5.4%)	89 (36.9%)
L03 IMMUNOSTIMULANTS	1	365	82 (22.5%)	30 (8.2%)	14 (3.8%)	11 (3%)	228 (62.5%)
L04 IMMUNOSUPPRESSANTS	28	24,017	9,841 (41%)	2,676 (11.1%)	1,563 (6.5%)	869 (3.6%)	9,068 (37.8%)
M01 ANTIINFLAMMATORY AND ANTIRHEUMATIC PRODUCTS	1	411	296 (72%)	22 (5.4%)	23 (5.6%)	14 (3.4%)	56 (13.6%)
M03 MUSCLE RELAXANTS	1	622	416 (66.9%)	62 (10%)	16 (2.6%)	8 (1.3%)	120 (19.3%)
M05 DRUGS FOR TREATMENT OF BONE DISEASES	2	617	436 (70.7%)	30 (4.9%)	21 (3.4%)	11 (1.8%)	119 (19.3%)
N01 ANESTHETICS	1	323	96 (29.7%)	43 (13.3%)	13 (4%)	7 (2.2%)	164 (50.8%)
N02 ANALGESICS	7	2,125	1,423 (67%)	178 (8.4%)	104 (4.9%)	69 (3.2%)	351 (16.5%)
N03 ANTIEPILEPTICS	1	102	47 (46.1%)	22 (21.6%)	4 (3.9%)	6 (5.9%)	23 (22.5%)
N05 PSYCHOLEPTICS	1	210	126 (60%)	22 (10.5%)	20 (9.5%)	7 (3.3%)	35 (16.7%)
N06 PSYCHOANALEPTICS	1	522	414 (79.3%)	50 (9.6%)	24 (4.6%)	6 (1.1%)	28 (5.4%)
N07 OTHER NERVOUS SYSTEM DRUGS	2	597	290 (48.6%)	68 (11.4%)	49 (8.2%)	20 (3.4%)	170 (28.5%)
R03 DRUGS FOR OBSTRUCTIVE AIRWAY DISEASES	4	2,344	850 (36.3%)	210 (9%)	174 (7.4%)	78 (3.3%)	1,032 (44%)
R06 ANTIHISTAMINES FOR SYSTEMIC USE	1	302	209 (69.2%)	18 (6%)	2 (0.7%)	2 (0.7%)	71 (23.5%)
R07 OTHER RESPIRATORY SYSTEM PRODUCTS	1	345	149 (43.2%)	42 (12.2%)	16 (4.6%)	5 (1.4%)	133 (38.6%)

**Figure 3:**
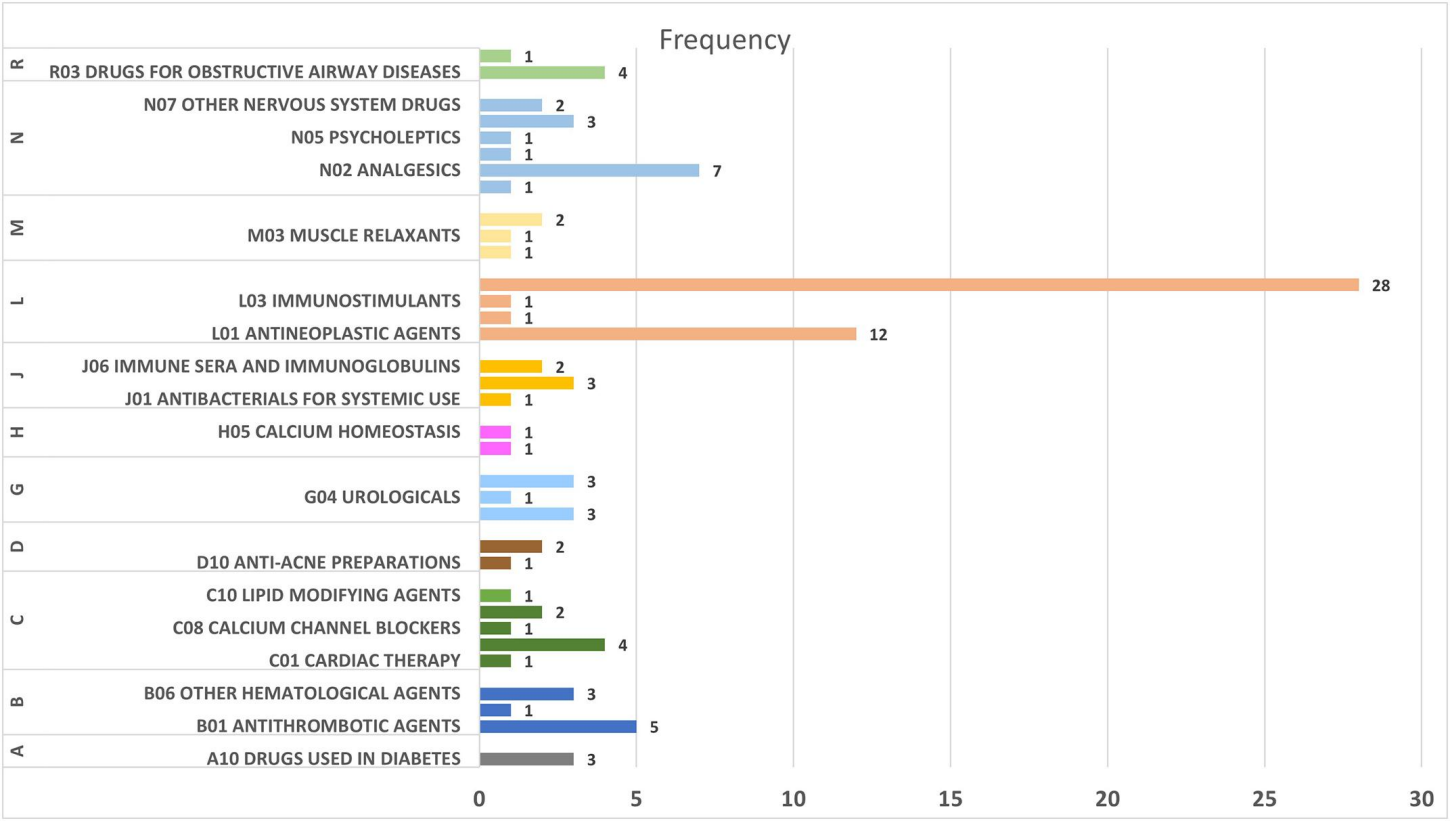
Distribution of drugs in different ATC categories associated with headache as ADR.

Figure 3 illustrates the distribution of drugs associated with headache as an ADR across different ATC categories. The largest number of drugs was found in the “L04 Immunosuppressants” category, with 28 drugs, followed by “B01 Antithrombotic Agents” with 5 drugs. Other categories, such as “J05 Antivirals for Systemic Use” and “N02 Analgesics,” also included several drugs linked to headache as an ADR. In contrast, categories like “B06 Other Hematological Agents” and “H03 Thyroid Therapy” contained only a single drug associated with this adverse effect.

## 4 Discussion

Our study reviewed data from the FAERS spanning from 2018 to 2024, providing a comprehensive overview of medications potentially associated with headache as an ADR. The refined dataset, consisting of 9,536,756 unique reports, highlights 313,166 cases where headache was reported as the primary suspected adverse event. We used disproportionality analysis by ROR and PRR to identify drugs with strong associations to headache.

### 4.1 Demographic Patterns in headache reports

Our findings showed that females constituted the majority of reports (66.66%), consistent with evidence that women have a 1.5- to 1.7-fold higher risk of adverse drug reactions than men (21–23). The potential contributing factors include sex-based differences in pharmacokinetics (e.g., hepatic metabolism, cytochrome P450 activity), pharmacodynamics, hormonal influences, and immunological responses, as well as higher medication use among women (24, 25).

The majority of headache cases in our study occurred in individuals aged 51–65 years, a population likely more susceptible to polypharmacy and chronic disease treatments, both known contributors to headache-related adverse drug reactions (4, 26). This aligns with evidence that elderly patients, particularly women, frequently experience headaches as ADRs, often linked to antivirals, antidepressants, and analgesics, with nearly half of cases classified as serious (4). Polypharmacy exacerbates this risk, as seen in primary headache disorders where comorbidities and prophylactic treatments drive high medication burdens (26, 27). However, the substantial proportion of missing data for age (36.57%) and sex (10.05%) suggests potential limitations in reporting completeness, which may influence the generalizability of these findings. Furthermore, the predominance of reports from the USA (65.87%) and Canada (12.06%) reflects the strength of North American pharmacovigilance systems, such as the FDA's FAERS, but may introduce geographic bias, underscoring the need for harmonized international data collection to enhance generalizability.

### 4.2 Associations with antivirals and other high-ROR drugs

Our analysis identified strong associations between headache and direct-acting antivirals (DAAs), particularly glecaprevir/pibrentasvir (ROR = 10.445) and sofosbuvir/velpatasvir (ROR = 9.729). This elevated reporting may reflect sampling bias from registration studies, where structured AE collection leads to higher frequencies than in routine clinical use, as evidenced by real-world data showing milder headache incidence (28–30). While DAAs like glecaprevir/pibrentasvir maintain high efficacy and tolerability, the consistency of headache reports across studies suggests a class effect warranting patient education and proactive management (31, 32).

### 4.3 Immunomodulatory drugs and headache frequency

Furthermore, our analysis revealed significant associations between headache and several medications, including eptinezumab-jjmr (ROR = 6.775), abaloparatide (ROR = 6.268), ledipasvir/sofosbuvir (ROR = 5.937), and galcanezumab-gnlm (ROR = 4.251). While these drugs serve distinct therapeutic purposes—from migraine prevention (eptinezumab, galcanezumab) to osteoporosis treatment (abaloparatide) and HCV therapy (ledipasvir/sofosbuvir)—headache emerges as a common adverse effect across these classes. Conversely, drugs like acetaminophen and cetirizine hydrochloride displayed low RORs (0.05 and 0.062, respectively), suggesting that these agents have a minimal association with headache. However, the elevated ROR for galcanezumab-gnlm (4.251) raises concerns about its long-term tolerability, despite favorable clinical trial safety profiles. The occurrence of headache as an ADR could indicate a failure of these medications to effectively prevent migraines. However, the elevated RORs for CGRP monoclonal antibodies like eptinezumab and galcanezumab may reflect indication bias, a known limitation of FAERS where drugs prescribed for headache-related conditions over-represent such reports, as noted by Musialowicz et al. (16). Excluding headache-related indications for bias control is challenging due to incomplete FAERS data, with elevated RORs likely reflecting migraine patient reporting patterns rather than causality. The high frequencies for these migraine-targeted mAbs in Table 2 likely arise from indication and sampling biases in registration trials, where headache monitoring is intensive, potentially over-representing events in FAERS compared to broader post-marketing surveillance (33).

Abaloparatide, an anabolic treatment for osteoporosis, exhibits a high ROR (6.268), which may reflect the occurrence of headache as an ADR. The drug demonstrates robust efficacy in increasing bone mineral density (BMD) in both men and postmenopausal women (34, 35). However, the randomized controlled trials (RCTs) of subcutaneous (SC) and transdermal formulations have reported headache as part of the safety profile, with the SC 80 μg dose showing a headache incidence of 16.1%, and the transdermal formulation showing an incidence of 9.9%.

Ledipasvir/sofosbuvir, a key hepatitis C therapy, achieved high sustained virologic response rates in cirrhotic patients, yet its high ROR (5.937) in our study may be linked to reported adverse effects like headache and fatigue (36).

Furthermore, our study findings demonstrated that immunomodulatory drugs, specifically apremilast, treprostinil, and adalimumab were frequently associated with headaches as an ADR. Apremilast, a PDE-4 inhibitor used in psoriasis and psoriatic arthritis (37), demonstrates a notable incidence of headache, particularly in the first two months of treatment, though symptoms often diminish over time (38). Similarly, treprostinil, an oral prostacyclin analogue for pulmonary arterial hypertension, commonly induces headache as a side effect, affecting nearly 70% of patients in clinical trials (39). Adalimumab, a TNF-α antagonist for Crohn's disease, also shows headache as a frequent ADR, though its benefits in maintaining remission often outweigh this risk (40).

The mechanisms underlying headache induction may differ among these drugs. Despite these variations, the consistent prevalence of headache across such therapies suggests that immunomodulatory agents, particularly those targeting chronic inflammatory conditions may inherently elevate headache risk. Prophylactic measures, such as melatonin or triptans for apremilast-associated headaches (38), or NSAIDs for acute cases, could mitigate this burden without compromising treatment efficacy.

### 4.4 ATC classification and risk profiles

The ATC classification analysis revealed that calcium homeostasis agents exhibited the highest risk ratio for headache (ROR = 6.268), suggesting a strong association between calcium-modulating drugs and headache induction. This finding aligns with existing literature demonstrating that both hypercalcemia (41) and hypocalcemia (42) are linked to headache pathophysiology, possibly due to role of calcium in neuronal excitability and vascular tone regulation. The drugs such as teriparatide, a calcium-regulating agent used in osteoporosis, were frequently associated with headache-related treatment discontinuation (43), further supporting the clinical relevance of this association. Similarly, calcium-based diagnostic tests, such as the calcitonin stimulation test, have been reported to induce headache as a common adverse reaction (44), reinforcing the notion that calcium homeostasis plays a critical role in headache generation. This high signal for calcium homeostasis agents (ROR = 6.268) may relate to their impact on neuronal excitability and vascular tone, as hypocalcemia or hypercalcemia disrupts cerebral blood flow, warranting monitoring in osteoporosis patients (41, 42). It is important to note that RORs indicate reporting disproportions rather than confirmed causal associations, necessitating further clinical validation. Similarly, systemic antivirals' association (ROR = 4.259) could stem from direct neuroinflammatory effects or interactions in chronic infections like HCV, though real-world studies confirm headaches are typically transient (45).

Moreover, systemic antivirals had a significantly higher risk ratio for inducing headaches (ROR = 4.259). This finding aligns with Musialowicz et al. (16), who identified antivirals (e.g., glecaprevir) and pulmonary hypertension medications (e.g., selexipag, epoprostenol) as having the strongest association with headaches (16).

### 4.5 Time-to-onset patterns by drug

Our findings highlighted that early-onset headaches (within 7 days of treatment initiation) are most frequently associated with ofatumumab, abaloparatide and fingolimod hydrochloride (78.6%, 69.5% and 58.5% of cases, respectively). This aligns with existing literature, as Zhou et al. (46) identified headache as a common adverse event linked to ofatumumab, often occurring within the first 30 days of therapy (46), while case reports describe persistent, new daily persistent headache-like symptoms in patients starting fingolimod (47). Similarly, apremilast and human immunoglobulin G were associated with early headache onset, corroborating studies showing apremilast-induced headaches typically emerging within the first month (48, 49) and IVIg-related cephalalgia often manifesting during or shortly after infusion (50, 51). Fingolimod-associated headaches often persists for months, resembling secondary headache disorders, yet they remain manageable in most cases without discontinuation (47). For IVIg, slower infusion rates and lower baseline blood pressure were identified as risk factors (51), implicating hemodynamic or immunological mechanisms.

On the other hand, drugs such as treprostinil and infliximab-dyyb, which showed a higher frequency of late-onset headache (occurring after more than 90 days of use), point to the possibility of delayed adverse reactions in patients. These patterns suggest the need for intensified early monitoring (≤7 days) for drugs like ofatumumab and fingolimod to mitigate acute risks, while long-term follow-up (>90 days) for agents like treprostinil could prevent delayed ADRs, informing personalized pharmacovigilance strategies. It may reflect distinct pathophysiological mechanisms associated with prolonged drug exposure. Clinical trials has reported that treprostinil, a prostacyclin analogue used in pulmonary arterial hypertension, was associated with persistent headache in 25%–34% of patients, often necessitating dose adjustments or discontinuation due to its vasodilatory effects (52, 53).

### 4.6 Time-to-onset patterns by ATC category

The ATC classification analysis revealed psychoanaleptics (N06) had the highest headache incidence (79.3% within 7 days of treatment initiation), followed by anti-inflammatory drugs (M01, 72%) and bone disease medications (M05, 70.7%). The psychoanaleptics such as amphetamine and dextroamphetamine are reported to cause headache with frequencies of 4% and 6% (54), respectively, further supporting the observed trend in our study. Similarly, a randomized controlled trial in ADHD children found Amphetamine Mixture—Dextroamphetamine Salts and Amphetamine Salts (Adderall) treatment, particularly at higher doses, increased headache severity per parental BSEQ reports, alongside other dose-dependent side effects like appetite loss and insomnia (55).

The association between anti-inflammatory drugs like ibuprofen and headache as an adverse drug reaction may be explained by medication-overuse headache, a disabling condition that contributes to chronic headache progression (56). The key risk factors for MOH include younger age (<50 years), female sex, anxiety/depression, physical inactivity, metabolic syndrome, and specific medications like tranquilizers and opioids (57–59). While ibuprofen showed a lower individual risk compared to triptans or opioids, dependency-like behaviors in MOH patients occur more frequently with opioid/triptan overuse than with NSAIDs.

### 4.7 Limitations and future directions

While this study offers valuable insights, it is important to acknowledge several limitations inherent in the use of the FAERS database, including the reliance on spontaneous reporting, which may lead to underreporting, particularly for mild side effects such as headaches compared to more severe ADRs. Additionally, the database lacks critical details such as dosage, duration of use, and patient comorbidities, potentially impacting causality assessment. Drugs indicated for headache treatment, such as CGRP monoclonal antibodies, may inflate reporting rates due to their therapeutic context, this circularity is unlikely to significantly bias results given the large dataset (9,536,756 reports) and diverse drug classes analyzed. Future research could address these gaps through clinical cohort studies or randomized controlled trials to better establish drug-headache associations. Although the study identifies commonly reported medications linked to headaches, the absence of consensus on the optimal data mining algorithm for ADR detection led to the selection of ROR due to its simplicity and higher sensitivity.

## 5 Conclusion

This comprehensive pharmacovigilance analysis underscores the importance of careful monitoring of drugs associated with headache as an ADR, especially those in high-risk categories such as immunosuppressants and systemic antivirals. The time-to-onset analysis highlights the need for surveillance strategies, with further mechanistic data needed for tailored approaches, particularly for drugs with a high likelihood of early-onset headaches. These findings contribute to a better understanding of the pharmacological profiles of drugs commonly used in clinical practice and inform monitoring strategies based on reporting patterns. Further research into the mechanisms underlying drug-induced headaches and the development of preventive measures is essential to optimize therapeutic outcomes and minimize adverse effects.

## Data Availability

The datasets presented in this study can be found in online repositories. The names of the repository/repositories and accession number(s) can be found below: https://fis.fda.gov/extensions/FPD-QDE-FAERS/FPD-QDE-FAERS.html.
